# Jinmaitong ameliorates diabetic peripheral neuropathy in streptozotocin-induced diabetic rats by modulating gut microbiota and neuregulin 1

**DOI:** 10.18632/aging.103750

**Published:** 2020-09-13

**Authors:** Jun Xie, Wei Song, Xiaochun Liang, Qian Zhang, Yue Shi, Wei Liu, Xiaohu Shi

**Affiliations:** 1 Department of Traditional Chinese Medicine, Peking Union Medical College Hospital, Peking Union Medical College, Chinese Academy of Medical Sciences, Beijing, China; 2 Medical Research Center, Peking Union Medical College Hospital, Peking Union Medical College, Chinese Academy of Medical Sciences, Beijing, China

**Keywords:** jinmaitong, diabetic peripheral neuropathy, diabetic complications, gut microbiota, neuregulin 1

## Abstract

Jinmaitong (JMT), a compound prescription of traditional Chinese medicine, has long been used as a therapy for diabetic peripheral neuropathy (DPN). However, the neuroprotective mechanisms of JMT and its effect on gut microbiota remained unknown. Here, we examined the effects of JMT on behavior, pathomorphology and gut microbiota in streptozotocin (STZ)-induced DPN rats. Compared to distilled water administration, JMT reversed decreases in mechanical withdraw threshold and intraepidermal nerve fiber density, improved neurological morphology of sciatic nerves, increased serum neuregulin 1 (NRG1) level and contactin-associated protein (Caspr)-positive paranodes, and decreased amyloid precursor protein (APP) accumulation in DPN rats. More importantly, JMT enriched nine species of the gut microbiota of DPN rats, helping to prevent dysbiosis. Among these species, p_Actinobacteria, p_Proteobacteria and c_*Actinobacteria* were negatively correlated with DPN phenotypes and positively correlated with serum NRG1 level. These results indicate that JMT may exert a neuroprotective effect by modulating phenotype-associated gut microbiota and increasing serum NRG1 level in STZ-induced DPN rats. JMT may therefore be an effective complementary and alternative anti-DPN therapy.

## INTRODUCTION

Type 2 diabetes mellitus (T2DM) is a common chronic disorder in older adults and poses a severe threat to their health [1]. Diabetic Peripheral Neuropathy (DPN), which occurs in 44% of older diabetics, is one of the most prevalent and devastating complications of diabetes [2]. DPN often presents earlier and more frequently than other diabetic complications, manifesting as a distal, symmetric, sensorimotor neuropathy with abnormal sensations such as paresthesias, allodynia, hyperalgesia, and spontaneous pain [3].

Currently, intensive glycemic control and symptomatic treatment are the only therapies available for DPN [4]. Although Neurotropin® (NTP), a non-protein extract from the inflamed skin of rabbits inoculated with vaccinia virus, is used to treat neuropathic pain and peripheral inflammation in DPN patients [5], it is not suitable as a long-term treatment due to the possibility of digestive and nervous systems side effects [6]. More effective treatments for DPN are therefore urgently needed.

Recent advancements in microbial sequencing, metagenomics, and bioinformatics have revealed an important relationship between gut flora and diabetes [7]. Evidence from multiple animal models and populations has confirmed that differences in gut microbiota composition between diabetic and healthy hosts contribute to various diabetic complications [8, 9]. However, few studies have investigated the role of gut microbiota on the development of DPN.

Drugs that act on gut microbiota have emerged as therapies for diabetes and its complications [10]. Since traditional Chinese medicine (TCM) is mainly delivered by oral administration, the natural compounds in TCM can interact extensively with the gut microbiota [11]. Several studies have demonstrated that TCM alleviates diabetes symptoms by preserving gut microbiota homeostasis. For example, TCM treatments increased levels of some short-chain fatty acid-producing bacteria and anti-inflammatory bacteria in the gut of diabetic hosts [12, 13].

Jinmaitong (JMT) is a compound prescription used in TCM to treat DPN. Our previous ultra-high performance liquid chromatography-quadrupole time-of-flight mass spectrometry (UPLC-QTOF-MS) analysis revealed that flavonoid and its glycosides, triterpenoids, and phenolic acids are the main constituents of JMT [14]. Several chemical components in JMT have been reported to be able to alleviate DPN both *in vivo* and *in vitro* [15–18]. Besides, the flavonoids and their glycosides identified in JMT were best known as strong antioxidants and could attenuate different kinds of neuropathic pain in behavioral, physiological, and biochemical tests [19]. Evidence from preclinical studies indicates that JMT improves nerve conduction velocity as well as pain and temperature sensation in DPN rats [20–22]. These results were confirmed in a double-blind randomized controlled trial, which demonstrated that JMT not only significantly improved blood glucose and lipid metabolism, and nerve conduction velocity in DPN patients, but also markedly ameliorated clinical symptoms such as cold, numbness, and pain of the extremities in DPN patients [22, 23]. However, these studies did not fully illustrate the therapeutic mechanism of JMT or the role of gut microbiota modulation in its neuroprotective effects.

In this study, we investigated the effects of JMT on gut microbiota in streptozocin (STZ)-induced DPN rats with gut dysbiosis. Fecal 16S rRNA gene sequencing was used to compare gut microbiota composition among normal control rats, distilled water-treated DPN rats, and JMT-treated DPN rats, so that to identify microbial species that contributed to the development of DPN or the neuroprotective effects of JMT. Data from our study deepen the understanding of the therapeutic mechanism of JMT on modulation of gut microbiota in DPN subjects and provide new evidence for the clinical application of the potential DPN therapy.

## RESULTS

### JMT alleviated peripheral neuropathy phenotypes in DPN rats

**Figure 1:**
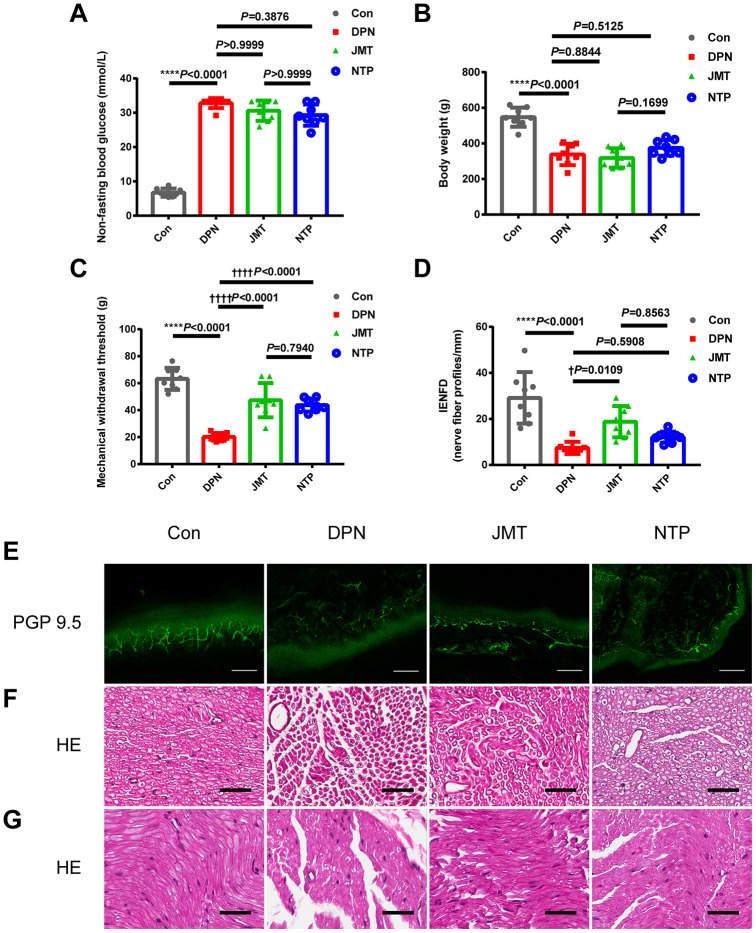
**Effects of JMT on phenotypes and histological morphology in DPN rats after 12 weeks.** (**A**) Non-fasting blood glucose level, (**B**) body weight, (**C**) mechanical withdraw threshold, (**D**) quantitation of intraepidermal nerve fiber density (IENFD) in different groups. (**E**) Representative images of PGP 9.5 (green) immunostaining of intraepidermal nerve fiber profiles at a magnification of 200×; scale bars, 100 μm. Representative HE staining photomicrographs of (**F**) cross-sections and (**G**) longitudinal sections from normal control rats, distilled water-treated DPN rats, JMT-treated DPN rats, and NTP-treated DPN rats at a magnification of 400×; scale bars, 50 μm. Means ± SD; n=3-8/group. ****p <0.0001 *vs.* normal control group; ††††*p* <0.0001, †*p* <0.05 *vs.* distilled water-treated DPN group. One-way ANOVA followed by Tukey’s multiple comparisons test or Kruskal-Wallis test followed by Dunn’s multiple comparisons test.

Twelve weeks after the induction of diabetes, STZ-injected rats displayed the peripheral neuropathy phenotypes characterized by increased non-fasting blood glucose level (Figure 1A, *p*<0.0001), decreased body weight (Figure 1B, *p*<0.0001), reduced Mechanical Withdraw Threshold (MWT) (Figure 1C, *p*<0.0001), and loss of intraepidermal nerve fibers in the hind paw (Figure 1D, E, *p*<0.0001). Both JMT and NTP treatment increased MWT (Figure 1C, *p*<0.0001) without affecting non-fasting blood glucose level and body weight in DPN rats (Figure 1A, 1B, *p*>0.05). In addition, JMT, but not NTP, improved intraepidermal nerve fiber density (IENFD) in DPN rats (*p*<0.05) compared to distilled water-treated DPN rats (Figure 1D, 1E, *p*>0.05). In summary, both JMT and NTP alleviated some peripheral neuropathy symptoms in DPN rats, with NTP showing no predominance than JMT.

### JMT improved the neurological morphology of sciatic nerves in DPN rats

**Figure 2:**
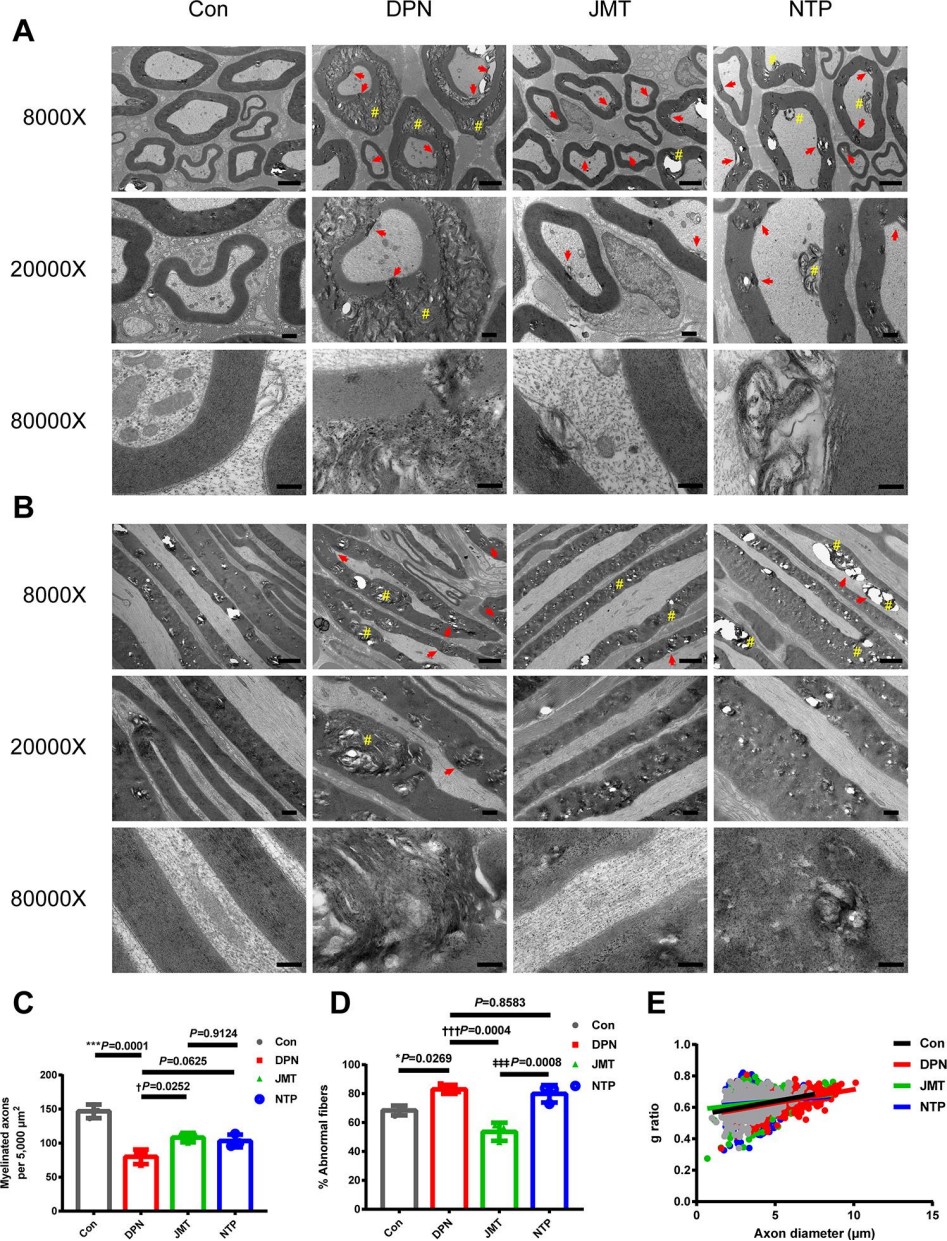
**Ultrastructure and morphometry of sciatic nerves under transmission electron microscopy.** Representative images of (**A**) cross-sections and (**B**) longitudinal sections of sciatic nerves from different groups at magnifications of 8000×, 20000×, and 80000×; scale bars, 2 μm, 500 nm, and 200 nm, respectively. Morphometric analyses of sciatic nerve showing (**C**) the number of myelinated axons per 5,000 μm^2^ and (**D**) the percentage of abnormal myelin fibers in different groups. Means ± SD; n = 3/group. (**E**) Quantification of g ratios for axons in different groups. n=632 axons from 3 normal control rats, n=525 axons from 3 distilled water-treated DPN rats, n=585 axons from JMT-treated DPN rats, n=544 axons from 3 NTP-treated DPN rats. ***p < 0.001, *p < 0.05, *vs.* normal control group; †††*p* < 0.001, †*p* < 0.05 *vs.* distilled water-treated DPN group; ‡‡‡*p* < 0.001 *vs.* JMT-treated DPN group. One-way ANOVA followed by Tukey’s multiple comparison test or Kruskal-Wallis test followed by Dunn’s multiple comparisons test. Pound signs (#) indicate onion-bulb and bubble form protrusions. Red arrows indicate lamellar separation between the axon and myelin sheath and demyelination.

Both hematoxylin and eosin (HE) staining (Figure 1F, 1G) and ultrastructural evaluation using a transmission electron microscope (TEM) (Figure 2A, 2B) showed that sciatic nerves from normal control rats contained myelinated fibers of varying diameters, regular contours, intact myelin sheaths, and thickness proportional to the diameter of their axons, which were uniformly distributed. In the distilled water-treated DPN group, myelinated fibers with axonal atrophy and myelin sheath deformation were observed and were sparsely distributed under the light microscopy (Figure 1F, 1G). Meanwhile, ultrastructure examinations revealed that myelin destruction had occurred as evidenced by onion-bulb and bubble form protrusions on the myelin sheath and axolemma border of myelinated axons. The most striking ultrastructural alterations of axonal myelin were vacuolization and lamellar separation, which consists of separated myelin fibers with large spaces between the axon and myelin sheath. Shrunken and swollen axons were also commonly observed, and both total damage and axonal deformations were present (Figure 2A, 2B). Qualitative assessment of ultrastructural characteristics revealed that the number of myelinated axons per 5000 μm^2^ (Figure 2C) decreased, while the percentage of abnormal myelin fibers with morphological alterations, including myelin infoldings, vacuolization, and uneven thickness increased in distilled water-treated DPN rats (Figure 2D).

Morphological abnormalities were ameliorated in both JMT-treated and NTP-treated DPN rats. The sciatic nerves of JMT-treated DPN rats contained myelin fibers of various sizes and with more proportional caliber sheaths surrounding the axon. Under light microscopy, myelin sheaths showed more regular contours with homogeneous distribution in JMT- and NTP-treated DPN rats compared with the distilled water-treated DPN rats (Figure 1F, 1G). JMT and NTP improved pathological ultrastructure features in myelin and axons to different degrees. Vacuolization and lamellar separation of axonal myelin were reduced to a greater extent after JMT treatment. Morphologic alterations and myelin breakdown were also reduced specifically in JMT-treated DPN rats, and deranged myelin sheath cells recovered after treatment (Figure 2A, 2B). Qualitative assessment of ultrastructural characteristics revealed that the number of myelinated axons per 5000 μm^2^ increased (Figure 2C, *p* < 0.05) and the percentage of abnormal myelin fibers decreased (Figure 2D, *p* < 0.001) in JMT-treated DPN rats, but not in NTP-treated DPN rats (Figure 2C, 2D, *p* > 0.05), compared to distilled water-treated DPN rats. Axons g ratios did not differ among different groups (Figure 2E, *p* > 0.05) [24], indicating that atrophy of myelinated fibers occurred to the same degree in both axons and myelin sheaths [25]. The observed g ratio around 0.6 may explain, at least in part, the preservation of baroreceptor function in the DPN rats [26].

### JMT prevented myelin and axonal damage and increased serum neuregulin 1 (NRG1) level in DPN rats

Demyelination and axonal degeneration are two key pathologic features of DPN. Myelin pathology is usually measured by paranodal architecture, while axonal damage is usually evaluated by measuring acute axonal damage indicator amyloid precursor protein (APP) levels [27, 28].

**Figure 3:**
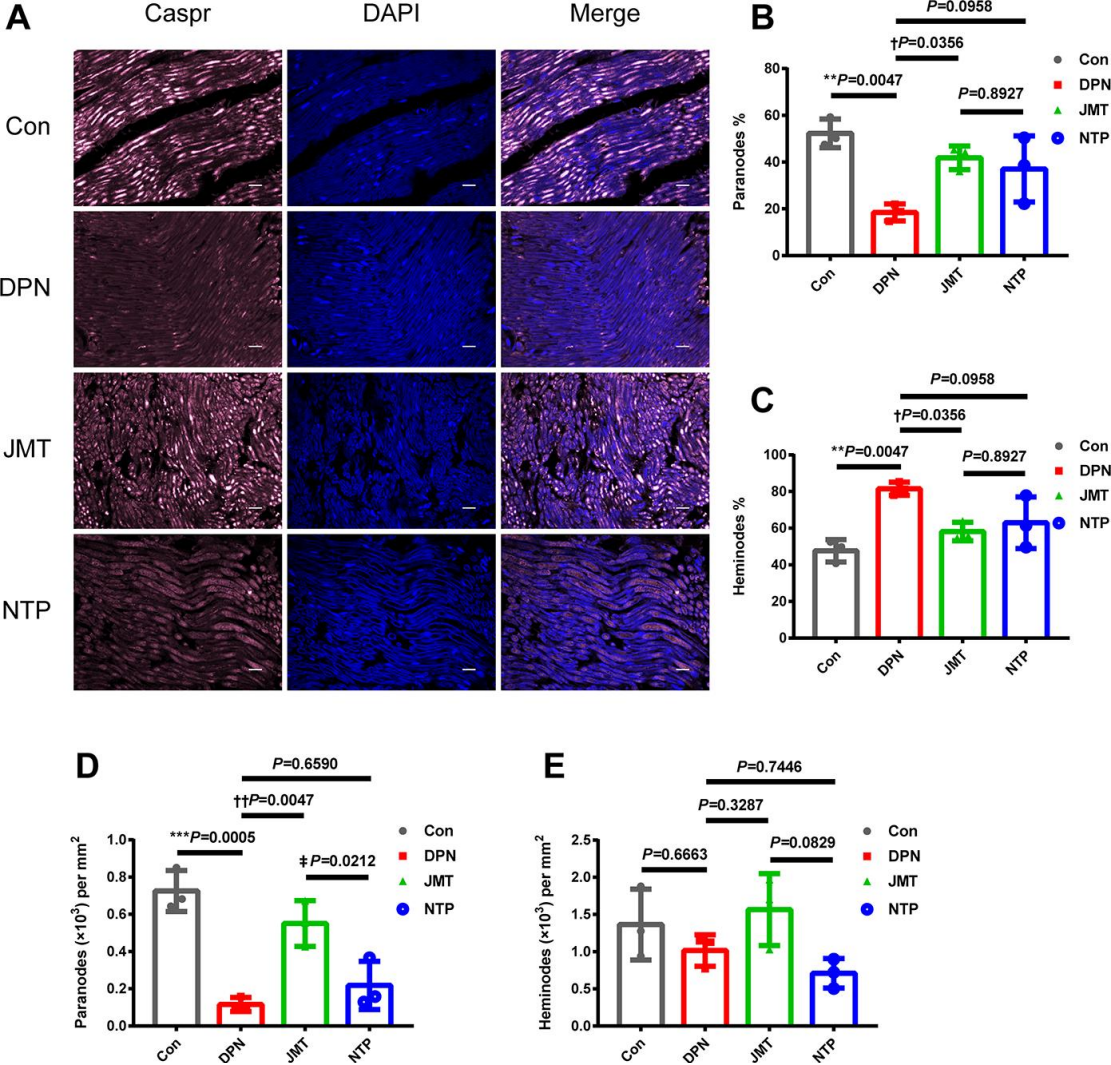
**JMT protected paranodal structures in the sciatic nerves of DPN rats.** (**A**) Representative images of longitudinal sections of sciatic nerves with immunolabeling for Caspr (pink) and counterstained with DAPI (blue, marking nuclei) at a magnification of 400×; scale bars, 20 μm. Percentage of (**B**) paranodes and (**C**) heminodes at paranodal junctions in the sciatic nerves of rats. Number of (**D**) paranodes and (**E**) heminodes per mm^2^ in the sciatic nerves of rats. Means ± SD; n=3/group. ***p < 0.001, **p < 0.01 *vs.* normal control group; ††*P* < 0.01, †*P* < 0.05 *vs.* distilled water-treated DPN group. ‡*p* < 0.05 *vs.* JMT-treated DPN group. One-way ANOVA followed by Tukey’s multiple comparison test.

Immunolabeling for contactin-associated protein (Caspr) at the paranodes is often used to quantify symmetrical paranodal densities and asymmetrical heminode densities, which are key indicators of structural damage and paranodal demyelination in peripheral nerves [29]. Here, immunostaining revealed that the number (Figure 3A, 3D, *p* < 0.001) and percentage (Figure 3A, 3B, *p* < 0.01) of Caspr-expressing paranodes were decreased, while heminode percentage was increased (Figure 3A, 3C, *p* < 0.01) in the sciatic nerves of distilled water-treated DPN rats compared to normal control rats. These changes were reversed by JMT treatment (Figure 3A–3D, *p* < 0.05), further suggesting that JMT protects myelin and paranodal architecture in DPN rats. In contrast, no differences were observed in these measures between NTP-treated and distilled water-treated DPN rats (Figure 3A–3D, *p* > 0.05). No significant differences in number of heminodes were observed among all the different groups (Figure 3E, *p* > 0.05), perhaps indicating that symmetrical paranodes were more severely damaged than asymmetrical heminodes in DPN rats.

**Figure 4:**
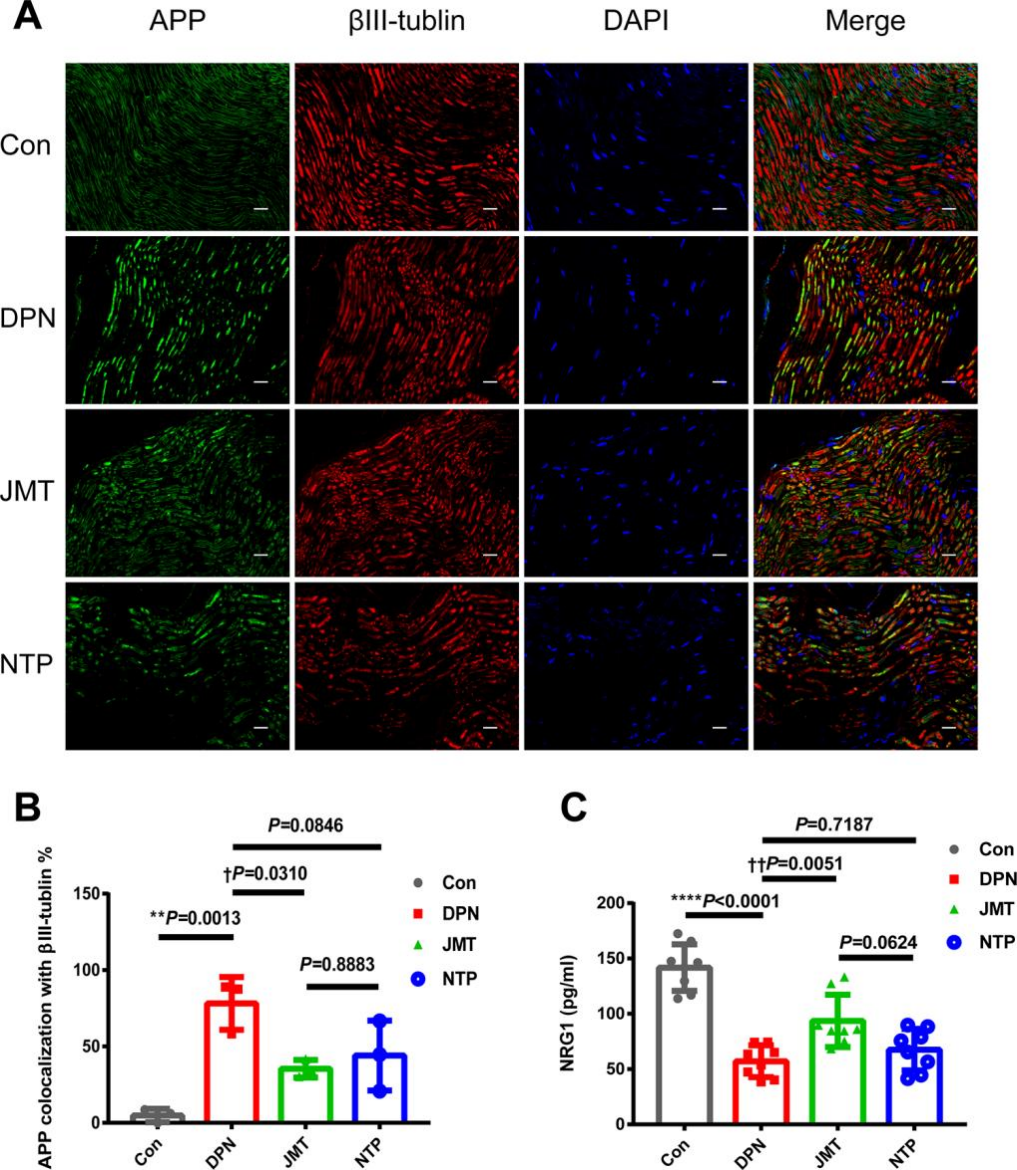
**JMT inhibited APP expression and promoted serum NRG1 level in DPN rats.** (**A**) Representative images of longitudinal sections of sciatic nerves with immunolabeling for APP (green), βIII-tubulin (red), and DAPI (blue, marking nuclei) at a magnification of 400×; scale bars, 20 μm. (**B**) Percentage of APP+ axonal area with βIII-tubulin colocalization. (**C**) Panels of serum NRG1 level in different groups. Means ± SD; n=3-8/group. ****p < 0.0001, **p < 0.01 *vs.* normal control group; ††*p* < 0.01, †*p* < 0.05 *vs.* distilled water-treated DPN group. One-way ANOVA followed by Tukey’s multiple comparison test.

Axonal damage was assessed by determining the percentage of APP and beta Tubulin 3 (βIII-tubulin) co-localized axons. APP+ axonal spheroids were clearly visible in longitudinal sections of sciatic nerves from distilled water-treated DPN rats, but not in normal control rats (Figure 4A, 4B, *p* < 0.01). The percentage of APP+ axonal spheroids was reduced in JMT-treated DPN rats compared to those treated with distilled water (Figure 4A, 4B, *p* < 0.05).

NRG1, a key promoter of neuronal survival, Schwann cell proliferation [30], and myelination [31], is crucial for peripheral nerve growth and development [32]. ELISA results showed that NRG1 level decreased in the serum of distilled water-treated DPN rats (Figure 4C, *p* < 0.0001), and JMT treatment reversed this effect (Figure 4C, *p*< 0.01).

Together, these data suggest that JMT exerted a neuroprotective effect against paranodal and axonal pathology with increased serum NRG1 level in DPN rats.

### JMT modulated microbiota composition in DPN rats

In order to further explore the mechanisms of JMT’s neuroprotective effects, fecal 16S rRNA gene sequencing and correlation analysis were used to identify differences in gut microbiota composition among normal control rats, distilled water-treated DPN rats, and JMT-treated DPN rats.

**Figure 5:**
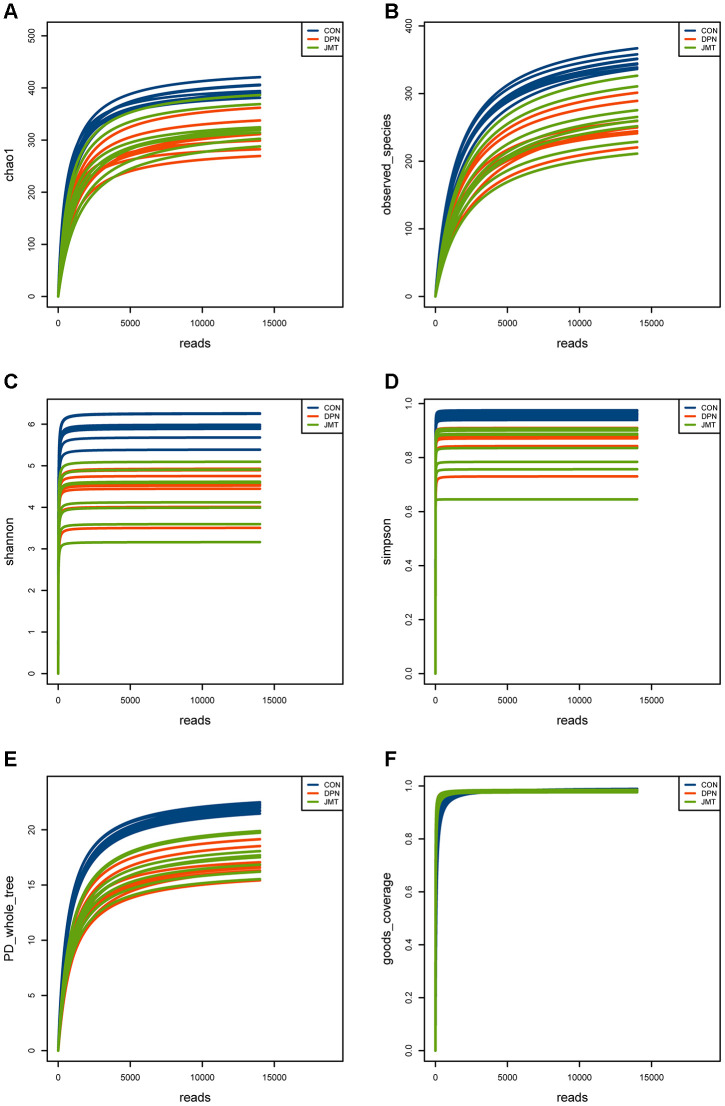
**Rarefaction curves of the alpha diversity indexes in different groups.** Rarefaction curves of (**A**) the Chao1 index, (**B**) observed species index, (**C**) Shannon index, (**D**) Simpson index, (**E**) PD whole tree index, (**F**) goods coverage index of fecal samples from different groups. All of the alpha diversity indexes achieved stability in different groups, which means the sequencing sample size for gut microbiota analysis is sufficient and the sequencing depth is enough.

As shown in Figure 5, the rarefaction curves of the alpha diversity indexes achieved stability which means the sequencing sample size for gut microbiota analysis is sufficient. Besides, the goods coverage index is very close to 1, which means the sequencing depth has basically covered all the species in the sample.

**Figure 6:**
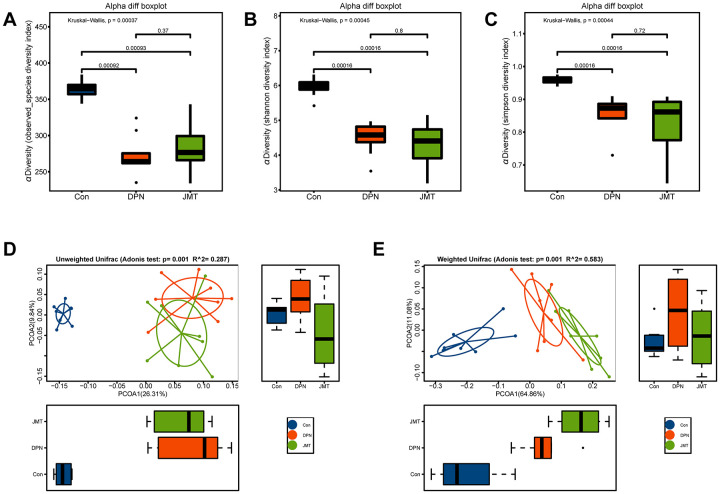
**JMT modulated microbiota composition in DPN rats.** Alpha diversity was evaluated by examining (**A**) the observed species diversity index, (**B**) Shannon index, and (**C**) Simpson index of fecal samples from different groups. Each box plot represents the median, interquartile range, minimum and maximum values. Principal coordinate analysis (PCoA) on (**D**) unweighted and (**E**) weighted UniFrac distances among different groups. Boxplots show the distribution of samples along the given axis, representing the median and interquartile range. Ellipses represent a 95% confidence interval around the cluster centroid. Clustering significance by body site was determined by Adonis (*p* < 0.001).

The observed species diversity index (Kruskal-Wallis, *p*=0.00092, Figure 6A), Shannon diversity index (Kruskal-Wallis, *p* = 0.00016, Figure 6B) and Simpson diversity index (Kruskal-Wallis, *p* = 0.00016, Figure 6C) analyses revealed the richness of bacterial colonies and community diversity was reduced in the fecal samples of distilled water-treated DPN rats, compared to normal control rats. However, no significant differences in these alpha diversity indexes were observed between distilled water-treated and JMT-treated DPN rats (Kruskal-Wallis, *p* > 0.05, Figure 6). Both unweighted (Adonis *p*=0.001, R^2^=0.287) and weighted (Adonis *p*=0.001, R^2^=0.583) UniFrac-based principal coordinates analysis (PCoA) revealed different microbiota composition clusters in normal control rats, distilled water-treated DPN rats, and JMT-treated DPN rats (Figure 6D, 6E), indicating significantly different beta diversities. Together, these alpha and beta diversity results suggest that JMT might regulate microbiota composition by increasing species diversity among taxa rather than within individual taxa.

**Figure 7:**
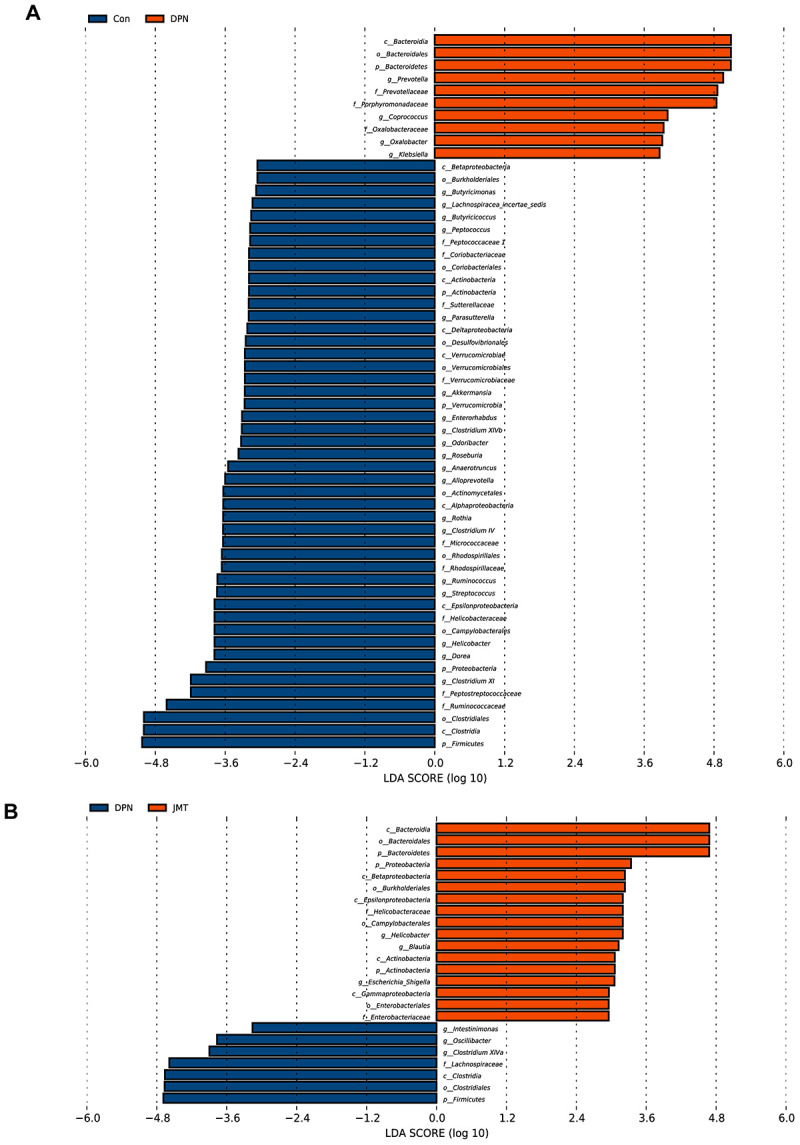
**Bacterial taxa differences among different groups.** Linear discriminant analysis coupled with effect size (LEfSe) analysis was performed to identify the bacterial taxa differentially represented in (**A**) normal control group *vs.* distilled water-treated DPN group rats and (**B**) distilled water-treated DPN group *vs.* JMT-treated DPN group rats at different taxonomy levels. LDA: linear discriminant analysis, n=8/group.

Linear discriminant analysis coupled with effect size (LEfSe) analysis was performed to identify the specific bacterial taxa differentially represented in normal control rats *vs.* distilled water-treated DPN rats (Figure 7A) and in distilled water-treated *vs*. JMT-treated DPN rats (Figure 7B). Compared with normal control rats, distilled water-treated DPN rats were enriched in Bacteroidetes at the phylum level, *Bacteroidia* at the class level, *Bacteroidales* at the order level, *Porphyromonadaceae*, *Prevotellaceae*, and *Oxalobacteraceae* at the family level, and *Klebsiella*, *Coprococcus*, *Prevotella*, and *Oxalobacter* at the genus level, which might be related to the pathogenesis of DPN (Figure 7A). However, we found that JMT treatment could counteract DPN-induced gut dysbiosis by enriching nine species which were also dominant in normal control rats (Figure 7). We therefore speculated that JMT ameliorated gut dysbiosis in distilled water-treated DPN rats by increasing the abundance of Actinobacteria and Proteobacteria at the phylum level, *Betaproteobacteria*, *Actinobacteria*, and *Epsilonproteobacteria* at the class level, *Burkholderiales* and *Campylobacterales* at the order level, *Helicobacteraceae* at the family level, and *Helicobacter* at the genus level (Figure 7).

### Association between gut microbiota abundance, DPN phenotypes, and serum NRG1 level

In order to verify the relationship between DPN pathogenesis and gut microbiota, we examined the Spearman’s correlations between alterations in microbiota composition and DPN phenotypes, such as body weight, non-fasting blood glucose level, mechanical withdraw threshold, intraepidermal nerve fiber density, and serum NRG1 level.

**Figure 8:**
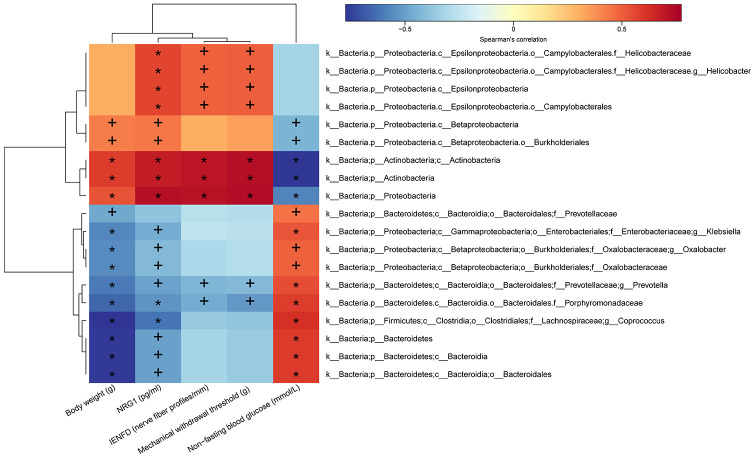
**Heatmap of correlations between gut microbiota abundance and DPN phenotypes and serum NRG1 level.** The intensity of the color represents the r-value of Spearman’s correlations (negative score, blue; positive score, red) between the relative abundance of the species and DPN phenotypes and serum NRG1 level, n=8/group, +*p* < 0.05, *p < 0.01.

As shown in Figure 8, all of the ten species that were enriched in distilled water-treated DPN rats were positively correlated with non-fasting blood glucose level (r > 0.45, *p* < 0.05), and negatively correlated with body weight (r < -0.45, *p* < 0.05). Besides, nine of them were also negatively correlated with serum NRG1 level (r < -0.40, *p* < 0.05); f_*Prevotellaceae* was not correlated with serum NRG1 level (r > -0.40, *p* > 0.05)*.* Among the nine species, f_*Porphyromonadaceae* and g_*Prevotella* were also negatively correlated with mechanical withdraw threshold (r < -0.40, *p* < 0.05) and intraepidermal nerve fiber density (r < -0.40, *p* < 0.05), indicating that these two species might also promote the pathogenesis of DPN. R- and *p*-values for these correlations are shown in Supplementary Tables 4 and 5. In contrast, the nine species enriched in both JMT-treated DPN rats and normal control rats were all positively correlated with serum NRG1 level (r > 0.40, *p* < 0.05). In addition, seven of those nine species were positively correlated with mechanical withdraw threshold (r > 0.50, *p* < 0.05) and intraepidermal nerve fiber density (r > 0.45, *p* < 0.05); c_*Betaproteobacteria* and o_*Burkholderiales* were not correlated with those measures (r <0.35, *p* > 0.05). Interestingly, among the seven species, p_Actinobacteria, p_Proteobacteria, and c_*Actinobacteria* were also positively correlated with body weight (r > 0.50, *p* < 0.01) and negatively correlated with non-fasting blood glucose level (r < -0.55, *p* < 0.01). These three species might therefore have contributed to JMT induced improvements in DPN phenotypes and symptoms of hyperglycemia and weight loss associated with increased serum NRG1 level (Figure 8, Supplementary Tables 4, 5).

## DISCUSSION

JMT is a traditional Chinese compound prescription developed by the Peking Union Medical College Hospital (PUMCH). It consists of 12 crude drug materials, including the seeds of *Cuscuta chinensis* Lam., the seeds of *Ligustrum lucidum* Ait., the herb of *Eclipta prostrata* L., the herb of *Prunella vulgaris* L., the seeds of *Litchi chinensis* Sonn., *Buthus martensii* K., the tender stem of *Cinnamomum cassia* Presl., the rhizoma of *Corydalis yanhusuo* W. T. Wang, the seeds of *Prunus persica* L., the seeds of *Cassia obtusifolia* L, the radix and rhizoma of *Asarum heterotropiodes* F., and *Hirudo nipponica* W [21]. In our previous study, we identified 72 chemical components of JMT using UPLC-QTOF-MS analysis, and found that it is composed mainly of flavonoid and its glycosides, triterpenoids, and phenolic acids [14]. Growing evidence indicates that these active components have anti-oxidative, anti-inflammation, anti-diabetes, and neuroprotective effects according to the pharmacology of Chinese Materia Medica and recent pharmacological researches [14].

JMT not only alleviated peripheral nerve injury as evidenced by improved mechanical withdrawal and warm thermal perception thresholds in STZ-induced DPN rats [20], but also ameliorated the clinical symptoms of DPN patients in double-blind, randomized clinical studies as evidenced by improved lipid metabolism and increased nerve conduction velocities [23]. Our previous *in vivo* experiments showed that JMT can inhibit DNA oxidative damage and apoptosis [22], as well as thioredoxin-interacting protein (TXNIP) and Nod-like receptor protein 3 (NLRP3) inflammasome activation [33] in the sciatic nerves of STZ-induced DPN rats. In the same rat DPN model, JMT also promoted the expression of nerve growth factors [20, 21], insulin-like growth factor 1 (IGF-1), and their receptors [14] in the sciatic nerves. Finally, our previous *in vitro* work showed that JMT and its active components promoted cell proliferation [17, 34] and autophagy [35, 36] and inhibited inflammation [15, 17] and apoptosis [37] in high-glucose cultured Schwann cells and dorsal ganglion neurons.

In this study, we investigated the neuroprotective effects of JMT in STZ-induced DPN rats, a widely used rat model of diabetes and diabetic complications [38]. Distilled water-treated DPN rats all developed classical DPN phenotypes characterized by hyperglycemia, decreased body weight, reduced MWT, and decreased IENFD. DPN rats also exhibited severely damaged myelin and axons in the sciatic nerves, which was accompanied by increased APP expression, and decreases in Caspr-expressing paranode percentages and serum NRG1 levels. These results indicated that hyperglycemia damaged the myelin and axon structures and inhibited their functions by inhibiting serum neurotrophic factor NRG1 expression, destroying Caspr-expressing paranodal structures, and promoting amyloid precursor protein accumulation in the sciatic nerves of distilled water-treated DPN rats.

Interestingly, JMT alleviated the DPN phenotypes by increasing both MWT and IENFD; NTP, which served as a positive control, increased MWT but not IENFD. Neither JMT nor NTP affected non-fasting blood glucose level or body weight in DPN rats, indicating that JMT and NTP may serve as useful adjuvants for antidiabetic drugs in DPN patients even if they cannot replace hypoglycemic drugs in clinical application.

In addition, morphological data from HE staining and TEM images indicated that JMT ameliorated the peripheral nerve damage in DPN rats by increasing axonal myelination and decreasing the percentage of abnormal fibers. The ELISA result showed that JMT rescued the serum NRG1 level of DPN rats. Therefore, we speculated that JMT might restore myelin structure by promoting serum NRG1 secretion, increasing the number and percentage of Caspr-positive paranodes, and inhibiting amyloid precursor protein accumulation in the sciatic nerves of DPN rats.

A growing number of studies have demonstrated important connections between gut microbiota and diabetic hosts [39], and gut microbiota dysbiosis can drive T2DM pathogenesis [40]. Consisted with the highly valued function of balanced microbiota compositions in T2DM, here, 16S rRNA gene sequencing revealed that distilled water-treated DPN rats also showed gut dysbiosis compared to normal control rats. LEfSe analysis identified ten enriched species in the distilled water-treated DPN group that are also abundant in diabetes patients [41, 42] and diabetic rodent models [43, 44], and are positively correlated with fasting blood glucose level [45]. Among them, f_*Porphyromonadaceae* and g_*Prevotella* were negatively correlated with body weight, IENFD, MWT and serum NRG1 level, indicating that these two species might promote the pathogenesis of DPN and inhibit NRG1 secretion. Other reports have shown that f_*Porphyromonadaceae* is enriched in diabetic-sensitive mice and associated with a high-fat diet-induced metabolic changes [46], while g_*Prevotella* is enriched in rats with advanced-stage type 1 diabetes [47]; these findings agree with our current results.

Interestingly, JMT reversed gut microbial dysbiosis by restoring nine species to levels observed in normal control rats. Though few reports have examined the relationship between these species and DPN or diabetes, some of them are beneficial in treating other intestinal diseases; *Helicobacteraceae* was effective against colitis-associated colorectal cancer [48], and the Gram-negative bacterium *Helicobacter* *pylori* helped prevent and treat inflammatory bowel diseases [49]. Among the nine species enriched in JMT-treated DPN rats, p_Actinobacteria, p_Proteobacteria, and c_*Actinobacteria* were positively correlated with body weight, IENFD, MWT, and NRG1 level, but negatively correlated with non-fasting blood glucose level. Therefore, we speculated that they may help ameliorate DPN phenotypes associated with increased NRG1 levels. Studies show that *Actinobacteria* also has beneficial effects in T2DM after Roux-en-Y gastric bypass [50] and in obesity patients on a high-fermentable fiber diet with inulin [51]. Some reports also suggest that *Actinobacteria* plays an antidepressant role in inflammatory models [52] and has anti-tumor effects [53]. These researches support the beneficial role of *Actinobacteria* in human health.

**Figure 9:**
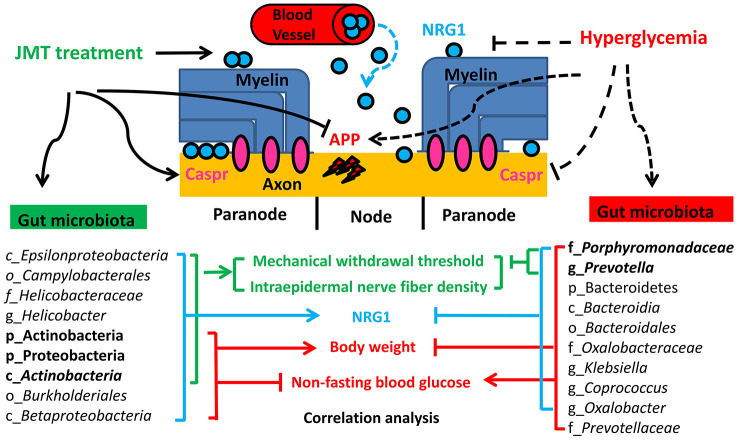
**Proposed mechanism for JMT-induced changes in gut microbiota composition that ameliorated DPN.**

Based on the studies and results discussed above, we hypothesize that JMT might ameliorate DPN phenotypes by enriching Actinobacteria and Proteobacteria at the phylum level and *Actinobacteria* at the class level; these microbiota abundances were positively correlated with body weight, MWT, IENFD, and serum NRG1 level and negatively correlated with non-fasting blood glucose level. By promoting serum NRG1 secretion in DPN rats, these taxa restored Caspr-positive paranodal structures, and decreased amyloid precursor protein accumulation in the sciatic nerves of DPN rats. They also increased the density of protein gene product 9.5 (PGP 9.5)-positive intraepidermal nerve fibers in the plantar skin and improved MWT in the sciatic nerves, and finally ameliorated hyperpathia in DPN rats. These changes might explain the JMT-induced reversal of DPN phenotypes and provide innovative insights on the underlying neuroprotective mechanisms of JMT (Figure 9).

This study identified differences in gut microbiota composition between DPN rats and normal control rats and to report that JMT exerts neuroprotective effects by modulating gut microbiota for the first time. The relationship between the identified bacterial taxa and DPN phenotypes and NRG1 levels provides new scientific evidence for the clinical application of JMT in treating DPN patients. However, some limitations of the present study should be addressed in future research. First, knockdown and overexpression models could be used to confirm the effects of genes and signaling pathways related to DPN and NRG1. Second, fecal transplants of the ‘pathogenic bacterias’ or ‘probiotics’ identified here could be applied in DPN rat models. Third, a combined multi-omics approach including both microbial metabolomics and proteomics might prove useful. Fourth, the screening of novel neuroprotective components from JMT should be taken into account in our future research. In the future, we plan on conducting comprehensive studies using transgenic mice, behavior studies, electrophysiology, genomics, transcriptomics, proteomics, metabonomics, and fecal transplants to further investigate the effects of JMT and screening the key effective components from JMT in diabetes and its complications.

## CONCLUSION

In summary, our results suggest that JMT treatment exerts protective effects on peripheral myelin by modulating gut microbiota associated with DPN phenotypes and increasing NRG1 level in STZ-induced DPN rats. These findings suggest that JMT might be useful as a clinical complementary and alternative treatment for DPN patients.

## MATERIALS AND METHODS

### Drug preparations

JMT is composed of the following 12 kinds of crude drugs: the seeds of *Cuscuta chinensis* Lam., the seeds of *Ligustrum lucidum* Ait., the herb of *Eclipta prostrata* L., the herb of *Prunella vulgaris* L., the seeds of *Litchi chinensis* Sonn., *Buthus martensii* K., the tender stem of *Cinnamomum cassia* Presl., the rhizoma of *Corydalis yanhusuo* W. T. Wang, the seeds of *Prunus persica* L., the seeds of *Cassia obtusifolia* L, the radix and rhizoma of *Asarum heterotropiodes* F., and *Hirudo nipponica* W. at a fixed ratio of 10:10:10:10:30:3:10:10:10:30:3:3. These drug materials were purchased from Beijing Tong Ren Tang Pharm Co., Ltd. (Beijing, China) and authenticated according to the Chinese Flora (http://frps.iplant.cn/frps/Uncaria) and Chinese Pharmacopoeia (Edition 2015, Volume I). Voucher specimens (No. jmt15A - jmt15L) were submitted to the Department of TCM, PUMCH, Beijing, China (Supplementary Table 1) [14]. The chemical profile of JMT was determined by UPLC/Q-TOF-MS analysis using the same formulation batch before use in these experiments; a total of 72 compounds were identified [14] (Supplementary Figure 1, Supplementary Table 2).

### Animals

All experiments were performed in accordance with the National Institutes of Health (NIH) “Guide for the Care and Use of Laboratory Animals, 8^th^ Edition” and were approved by the Animal Ethics Committees of PUMCH [certificate no. XHDW-2018-009]. Specific Pathogen Free (SPF) Sprague Dawley rats (6-weeks old, 160-200 g) were purchased from Vital River (Beijing, China). Upon arrival at the vivarium, rats were quarantined and monitored for one week. Rats were socially housed in SPF clean level conditions using standard rat cages and conventional bedding. Rats were given access to food and water ad libitum. All animal rooms were climate controlled (22–26°C, 40–70% humidity) with a 12-h light/dark cycle [54]. Diabetes was induced by administering freshly dissolved STZ (55 mg/kg in 0.1 mol/L citrate buffer, pH 4.5, i.p.) following an overnight fast. Hyperglycemia was verified 3 days post-STZ (ACCU-CHEK Active; Roche, Basel, Switzerland); rats with non-fasting blood glucose levels ≥ 16.7 mmol/L were classified as diabetic. Diabetic rats were randomly assigned to one of three groups (distilled water-treated DPN rats, JMT-treated DPN rats, or NTP-treated DPN rats; all n=8); age-matched healthy rats were assigned to the normal control group. Rats received 13.9 g/kg/d of crude drug of JMT, 1.6 NU/kg/d of NTP, or 10 mL/kg/d of distilled water daily. JMT and NTP dosages were determined by adjusting the clinical dosage for human adults by a factor equal to the human to rat body surface area ratio. Animals were monitored daily and maintained for 12 weeks post-STZ.

### Mechanical withdraw threshold

MWT was assessed 12 weeks post-STZ using the Von Frey Pain Measurement Instrument (cat. no. 2391; IITC Life Science, Woodland Hills, CA, USA). Rats were individually placed in a clear plastic cage containing mesh (1 cm^2^ perforations). After a 15-min acclimation period, the middle of the plantar of each rat was vertically stimulated with the electronic Von Frey probe, making it appear slightly S-shaped, and the paw withdrawal response was observed. A quick flinching reaction immediately after stimulation was considered a positive reaction, and pressure values (grams) were recorded. A paw withdrawal reaction caused by physical activity was not reported as positive. Test was repeated three times at the interval of 15 min and mean value was finally recorded [55].

### Blood collection and tissue processing

Blood samples were collected from the abdominal aorta and the serum was stored at -80°C. The bilateral sciatic nerves and plantar skin were cut with sharp scissors and immediately rinsed with ice-cold phosphate buffered saline (PBS) immediately. The sciatic nerve was isolated and cut into two segments: one segment was fixed in 4% paraformaldehyde (PFA) with 2.5% glutaraldehyde in 0.1 M PBS, pH 7.4 at 4°C overnight for ultrastructure observation; the other segment of sciatic nerve and plantar skin were fixed in 4% PFA for pathological and immunohistochemical staining. Rats were sacrificed by cervical dislocation after tissue processing.

### Enzyme-linked immunosorbent assay (ELISA)

Serum NRG1 level was measured and calculated using a commercially available kit (Cloud-Clone, Houston, TX, USA) according to the manufacturer’s instructions. Standard or sample, detection reagent, substrate solution and stop solution were sequentially added to wells and incubated with repeated washing as appropriate. Optical density at 450 nm was immediately measured with a plate reader, and sample values were then calculated from the standard curve.

### Electron microscopy and morphometric analysis

The 4% PFA with 2.5% glutaraldehyde fixed sciatic nerve tissues were sent to the Electron Microscopy Center in the Centralab Institute of Basic Medical Sciences at the Chinese Academy of Medical Sciences for ultrastructure observation. The nerves were rinsed in PBS, postfixed in 1% osmium tetroxide, embedded in epon, and dehydrated with ethanol and acetone. Ultrathin sections (70 nm) were stained with uranyl acetate and lead citrate and imaged in a transmission electron microscope (TEM-1400, JEOL, Tokyo, Japan) at 8000×, 20000×, and 80000× magnifications. Morphometric parameters such as number of myelinated axons per 5000 μm^2^, percentage of abnormal fibers (fibers with irregular shapes, infoldings, or compacted myelin), and g ratio values (the ratio of inner axon diameter to outer diameter of the myelin sheath, which was used to assess axonal myelination) were analyzed by EM imaging software (RADIUS, EMSIS, Münster, Germany) [56].

### Immunohistochemistry

Sciatic nerve tissues fixed in 4% PFA were embedded in paraffin, and cut into 4 μm sections. Sections were stained with hematoxylin and eosin and imaged using an ultra-compact image capture device (Aperio CS2, Leica, Wetzlar, Germany) at 400× magnification. The remaining sciatic nerve sections were treated as follows: regular dewaxing, antigens repair with hot citric acid buffer (pH 6.0), blocking with 10% goat serum. The sections were then incubated overnight with the following primary antibodies at 4°C: Caspr, APP, and βIII-tubulin, followed by the appropriate fluorophore-conjugated secondary antibodies for 40 min at room temperature. Detailed primary and secondary antibody information is shown in Supplementary Table 3. The sections were then washed in PBS and coverslipped in glycerin mounting medium with DAPI. Images were captured using a Nikon A1R confocal system (A1R, Nikon, Tokyo, Japan) at 400× magnification. Quantitative analyses were carried out using Nikon NIS-Element Analyzer.

### Intraepidermal nerve fiber density

Fixed plantar skin tissues were washed in PBS containing increasing amounts of sucrose. After washing, the samples were snap-frozen in optimum cutting temperature compound and stored at -80°C. Three longitudinal 50-μm-thick plantar skin sections were blocked with 3% goat serum containing 0.5% porcine gelatin and 0.5% Triton X-100 in Super-Block blocking buffer (Thermo Scientific, Rockford, IL) at room temperature for 2 h. The sections were then incubated overnight with PGP 9.5 at 4°C and then incubated with Alexa Fluor 488 goat anti-rabbit secondary antibody at room temperature for 1 h. Detailed antibody information is shown in Supplementary Table 3. Sections were then coverslipped with glycerin mounting medium. Representative images of intraepidermal nerve fibers were obtained using a Nikon A1R confocal system at 200× magnification. Numbers of intraepidermal nerve fiber profiles and epidermis lengths were assessed using Nikon NIS-Element Analyzer. IENFD was calculated as the number of nerve fiber profiles per millimeter of epidermis [57].

### DNA extraction and 16S rRNA gene sequencing

Fecal samples were harvested after an overnight fast at week 12 and then immediately frozen in liquid nitrogen and stored at -80°C. Genomic DNA was extracted from each fecal sample using the E.Z.N.A.^®^ Stool DNA Kit (OMEGA, Norcross, GA, USA) combined with bead beating according to the manufacturer’s instructions. The 16S rRNA gene sequencing procedure was performed by the Realbio Genomics Institute (Shanghai, China). Extracted genomic DNA was used as the template to amplify the V3-V4 regions of 16S rRNA genes. Amplicon libraries were quantified using a Qubit 2.0 Fluorometer (Thermo Fisher Scientific, Waltham, US) and then sequenced on the Illumina HiSeq platform (Illumina, San Diego, US) with paired-end reads of 250 bp. After discarding singleton reads and removing chimeras, tags were clustered into operational taxonomic units using USEARCH (v7.0.1090) at 97% similarity. A representative sequence of each operational taxonomic unit was then subjected to the taxonomy-based analysis using the RDP database. Heatmap was created using R. Cluster analysis. Alpha diversity and beta diversity were analyzed using QIIME. LEfSe was performed to identify bacterial taxa differentially represented among the groups at different taxonomy levels [58].

### Statistical analysis

GraphPad software (San Diego, CA, USA) was used for statistical analysis and graph generation. Data are expressed as mean ± standard deviation unless otherwise indicated. Normally distributed data were analyzed using one-way ANOVA with Tukey’s test for multi-group independent samples. Non-normally distributed data were analyzed using the Kruskal-Wallis test followed by Dunn’s multiple comparisons test for multi-group independent samples. Statistical significance was defined by *p* < 0.05.

## Supplementary Material

Supplementary Figure 1

Supplementary Table 1

Supplementary Table 2

Supplementary Table 3

Supplementary Table 4

Supplementary Table 5
