# Supplementary material for: Jinmaitong ameliorates diabetic peripheral neuropathy in streptozotocin-induced diabetic rats by modulating gut microbiota and neuregulin 1
Source: Aging (Albany NY). 2020 Sep 13;12(17):17436–58. doi: 10.18632/aging.103750 (PMC7521543; doi:10.18632/aging.103750)
Supplement: Supplementary Figure 1 [file aging-12-103750-s004..pdf]

SUPPLEMENTARY FIGURE

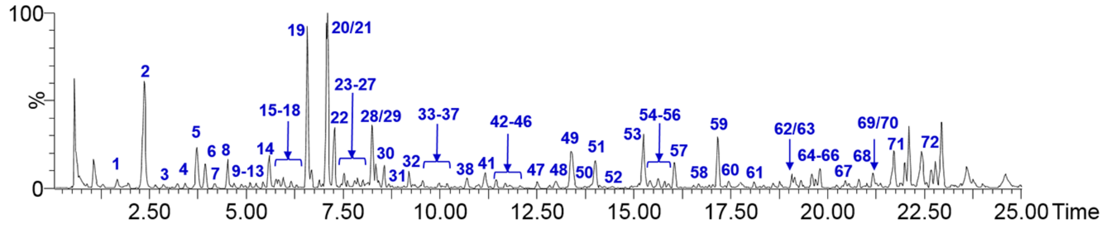

Supplementary Figure 1. Base peak ion chromatogram of JMT in negative scan. A figure from our previous published study using the same batch of JMT drug is provided for reference (W. Song et al., Jinmaitong, a Traditional Chinese Compound Prescription, Ameliorates the Streptozocin-Induced Diabetic Peripheral Neuropathy Rats by Increasing Sciatic Nerve IGF-1 and IGF-1R Expression, Frontiers in Pharmacology 10 (2019) 255. doi: 10.3389/fphar.2019.00255).
