# Supplementary material for: Jinmaitong ameliorates diabetic peripheral neuropathy in streptozotocin-induced diabetic rats by modulating gut microbiota and neuregulin 1
Source: Aging (Albany NY). 2020 Sep 13;12(17):17436–58. doi: 10.18632/aging.103750 (PMC7521543; doi:10.18632/aging.103750)
Supplement: Supplementary Table 1 [file aging-12-103750-s006..pdf]

SUPPLEMENTARY TABLE

Supplementary Table 1. Detailed information of the crude drugs in JMT.

No.	Drug Name	Authentication	Voucher specimen
jmt15-A	Semen Cuscutae	seeds of *Cuscuta chinensis* Lam.	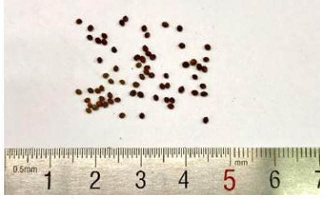
jmt15-B	Fructus Ligustri lucidi	seeds of *Ligustrum lucidum* Ait.	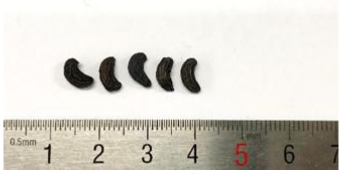
jmt15-C	Herba Ecliptae	whole herb of *Eclipta prostrata* L.	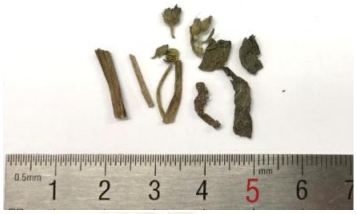
jmt15-D	Herba Prunella vulgaris	whole herb of *Prunella vulgaris* L.	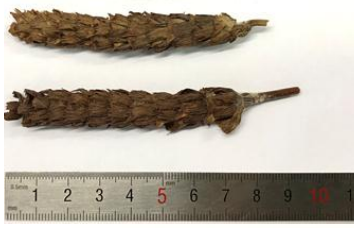
jmt15-E	Semen Litchi	seeds of *Litchi chinensis* Sonn.	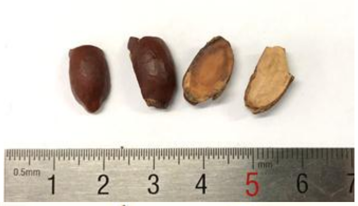
jmt15-F	Scorpio	*Buthus martensii* K.	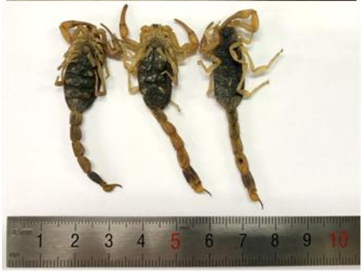
jmt15-G	Ramulus Cinnamoml	tender stem of *Cinnamomum cassia* Presl.	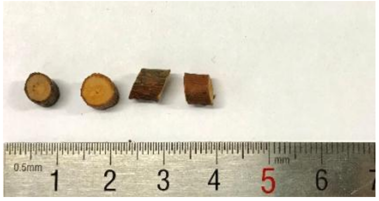

jmt15-H	Rhizoma Corydalis	rhizoma of *Corydalis yanhusuo* W. T. Wang	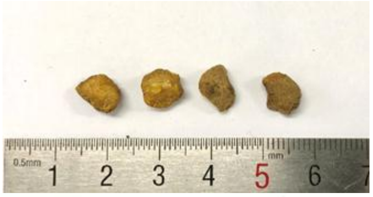
jmt15-I	Semen Persicae	seeds of *Prunus persica* L.	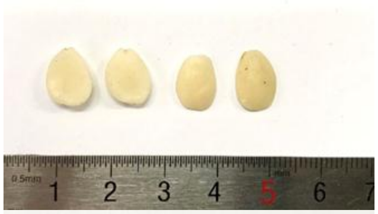
jmt15-J	Semen Cassiae	seeds of *Cassia obtusifolia L.* or *Cassia tora* L.	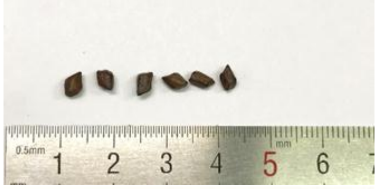
jmt15-K	Radix et Rhizoma Asari	radix and rhizoma of *Asarum heterotropiodes* F.	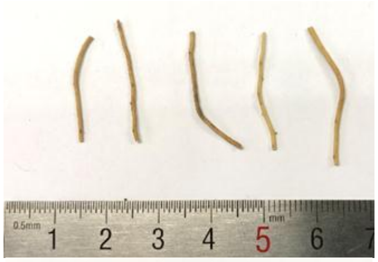
jmt15-L	Hirudo	*Hirudo nipponica* W.	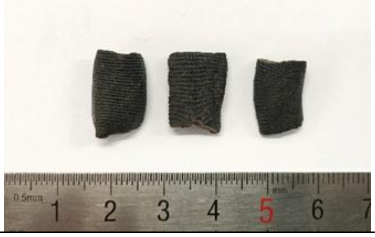

Note: A table from our previous published study using the same batch of JMT drug is provided for reference (W. Song et al., Jinmaitong, a Traditional Chinese Compound Prescription, Ameliorates the Streptozocin-Induced Diabetic Peripheral Neuropathy Rats by Increasing Sciatic Nerve IGF-1 and IGF-1R Expression, Frontiers in Pharmacology 10 (2019) 255. doi: 10.3389/fphar.2019.00255).
