# Supplementary material for: Jinmaitong ameliorates diabetic peripheral neuropathy in streptozotocin-induced diabetic rats by modulating gut microbiota and neuregulin 1
Source: Aging (Albany NY). 2020 Sep 13;12(17):17436–58. doi: 10.18632/aging.103750 (PMC7521543; doi:10.18632/aging.103750)
Supplement: Supplementary Table 2 [file aging-12-103750-s003..docx]

**Supplementary Table 2. Characterization of chemical constituents in JMT by UPLC-QTOF-MS analysis.**

| Peak  No. | t_R_  (min) | Measured  [M­-H]^-^ (m/z) | Predicted  [M­-H]^-^ (m/z) | Δ (ppm) | Formula | (–)­MS^E^ (m/z) | Identification | Derived  From |
| --- | --- | --- | --- | --- | --- | --- | --- | --- |
| 1 | 1.72 | 169.0130 | 169.0137 | -4.14 | C_7_H_5_O_5_ | 125.0235, 83.0116 | gallic acid | D |
| 2 | 2.36 | 515.1127 | 515.1190 | -12.23 | C_25_H_24_O_12_ | 353.1021, 191.0067 | isochlorogenic acid C | C |
| 3 | 2.79 | 167.0334 | 167.0342 | -4.79 | C_8_H_8_O_4_ | 135.0817 | vanillic acid | D |
| 4 | 3.98 | 153.0180 | 153.0188 | -5.23 | C_7_H_6_O_4_ | 109.029 | protocaechuic acid | E |
| 5 | 3.73 | 353.0997 | 353.1025 | -7.93 | C_20_H_18_O_6_ | 339.0869, 310.0527 | asarinin | J |
| 6 | 3.85 | 633.3972 | 633.4003 | -4.89 | C_36_H_58_O_9_ | 471.3481, 453.3340 | ecliptasaponin A or D | C |
| 7 | 4.23 | 633.3977 | 633.4003 | -4.10 | C_36_H_58_O_9_ | 471.3482, 453.3339 | ecliptasaponin A or D | C |
| 8 | 4.50 | 289.0735 | 289.0712 | 7.96 | C_15_H_14_O_6_ | 245.0803, 205.0510, 179.0345 | epicatichin | E |
| 9 | 4.53 | 919.2710 | 919.2719 | -0.98 | C_39_H_52_O_25_ | 271.0615, 256.0369 | cassiaside B2 | I |
| 10 | 4.98 | 685.2333 | 685.2344 | -1.61 | C_31_H_42_O_17_ | 523.1820, 453.3307 | specnuezhenide | B |
| 11 | 5.10 | 313.0352 | 313.0348 | 1.28 | C_16_H_10_O_7_ | 313.0350, 298.1503 | wedelolactone | C |
| 12 | 5.29 | 609.1882 | 609.1819 | 10.34 | C_28_H_34_O_15_ | 447.1285, 285.0753 | hesperidin | E |
| 13 | 5.47 | 901.2633 | 901.2614 | 2.11 | C_39_H_50_O_24_ | 253.0511 | emodin-1-O-β-D-tetrapyranoglucoside | I |
| 14 | 5.58 | 283.0221 | 283.0243 | -7.77 | C_15_H_8_O_6_ | 255.0287, 239.0338, 211.0173 | rhein | I |
| 15 | 5.77 | 353.0856 | 353.0873 | -4.81 | C_16_H_18_O_9_ | 191.0565, 112.9657 | chlorogenic acid | A,C |
| 16 | 5.80 | 957.5075 | 957.5059 | 1.67 | C_48_H_78_O_19_ | 795.4522, 455.3437 | ecliptasaponin B or III | C |
| 17 | 6.00 | 563.1412 | 563.1401 | 1.95 | C_26_H_28_O_14_ | 473.1082, 443.0975, 383.0764 | schaftoside | E |
| 18 | 6.16 | 340.1535 | 340.1549 | -4.12 | C_20_H_23_NO_4_ | 178.0552, 163.0738 | tetrahydrojatrorrhizine | G |
| 19 | 6.62 | 595.1617 | 595.1663 | -7.73 | C_27_H_32_O_15_ | 271.0625, 255.0403 | rubrofusarin-6-o-β-gentiobioside | I |
| 20 | 7.09 | 463.0883 | 463.0877 | 1.30 | C_21_H_20_O_12_ | 463.0882, 301.0350 | hyperoside | A |
| 21 | 7.15 | 419.1029 | 419.0978 | 12.17 | C_20_H_20_O_10_ | 257.0465 | cassiaside | I |
| 22 | 7.28 | 463.0888 | 463.0877 | 2.38 | C_21_H_20_O_12_ | 463.0885, 301.0347 | isoquercitrin | A,C,D |
| 23 | 7.51 | 253.0515 | 253.0501 | 5.53 | C_15_H_10_O_4_ | 225.0110, 149.0025 | chrysophanol | C |
| 24 | 7.65 | 491.1178 | 491.1190 | -2.44 | C_23_H_24_O_12_ | 329.0618, 298.0155 | hesperidin-6-o-β-D-glucoside | I |
| 25 | 7.73 | 447.0923 | 447.0927 | -0.89 | C_21_H_20_O_11_ | 285.0411, 175.0387, 133.0280 | luteoline 7­O­glucoside | A,D |
| 26 | 7.83 | 465.1020 | 465.1033 | -2.80 | C_21_H_22_O_12_ | 303.0502， 285.0471 | dihydroquercetin | B |
| 27 | 8.05 | 795.4479 | 795.4531 | -6.54 | C_42_H_68_O_14_ | 841.4561, 633.3463, 453.3351 | ecliptasaponin I,C,IV or XV | C |
| 28 | 8.23 | 447.0932 | 447.0927 | 1.12 | C_21_H_20_O_11_ | 285.0395, 175.0386, 151.0023 | luteoloside | I |
| 29 | 8.23 | 445.0787 | 445.0771 | 3.59 | C_21_H_18_O_11_ | 269.0461, 151.0023, 117.0315 | apigenin-7-O-glucronide | J |
| 30 | 8.52 | 447.0934 | 447.0927 | 1.57 | C_21_H_20_O_11_ | 285.0404, 175.0388, 133.0281 | astragalin | A |
| 31 | 9.06 | 565.1522 | 565.1558 | -6.37 | C_39_H_52_O_25_ | 271.0609, 256.0385 | cassiaside B | I |
| 32 | 9.25 | 685.2346 | 685.2344 | 0.29 | C_31_H_42_O_17_ | 523.1809, 453.3298 | nuezhenoside | B |
| 33 | 9.50 | 785.2531 | 785.2504 | 3.44 | C_35_H_46_O_20_ | 623.1957, 477.1320, 299.0743 | echinacoside | B |
| 34 | 9.64 | 843.4295 | 843.4378 | -9.84 | C_42_H_68_O_17_ | 841.4507, 633.3500, 471.3392 | ecliptasaponin VI | C |
| 35 | 9.98 | 623.1966 | 623.1976 | -1.60 | C_29_H_36_O_15_ | 461.1445, 153.0761 | acteoside | B |
| 36 | 10.12 | 359.0758 | 359.0767 | -2.51 | C_18_H_16_O_8_ | 322.1003 | rosmarinicacid | D |
| 37 | 10.25 | 269.0463 | 269.0450 | 4.83 | C_15_H_10_O_5_ | 241.0471, 213.0545,185.086 | emodin | G |
| 38 | 10.60 | 299.1139 | 299.1131 | 2.67 | C_14_H_20_O_7_ | 137.0587, 119.0495 | salidroside | B |
| 39 | 10.96 | 1071.3639 | 1071.3557 | 7.65 | C_48_H_64_O_27_ | 1117.2851, 909.2393, 685.5023 | oleonuezhenide | B |
| 40 | 11.00 | 453.3335 | 453.3369 | -7.50 | C_30_H_46_O_3_ | 437.3401, 411.3272 | 3-hydroxy oleanolic acid | C |
| 41 | 11.16 | 301.0339 | 301.0348 | -2.99 | C_15_H_10_O_7_ | 181.0131, 165.9892, 119.0493 | quercetin | A,C,D |
| 42 | 11.50 | 505.1331 | 505.1346 | -2.97 | C_24_H_26_O_12_ | 343.0829, 313.0352 | cassiin | I |
| 43 | 11.63 | 449.1073 | 449.1084 | -2.45 | C_21_H_22_O_11_ | 287.0547, 151.0025, | eriodictyol-7-o-glucoside | J |
| 44 | 11.67 | 451.1072 | 451.1088 | -3.55 | C_17_H_24_O_14_ | 407.2264, 375.0891 | privet acid | B |
| 45 | 11.86 | 1071.3564 | 1071.3557 | 0.65 | C_48_H_64_O_27_ | 909.3015, 685.1958, 299.0939 | nuezhenide G13 | B |
| 46 | 12.00 | 368.1875 | 368.1862 | 3.53 | C_22_H_27_NO_4_ | 336.1043, 320.1274, 294.0861 | corydaline | G |
| 47 | 12.52 | 279.2330 | 279.2324 | 2.15 | C_18_H_32_O_2_ | 279.2329 | linoleic acid | H |
| 48 | 13.04 | 283.0623 | 283.0607 | 5.65 | C_16_H_12_O_5_ | 268.0368, 240.0397 | obtusifolin | I |
| 49 | 13.38 | 285.0370 | 285.0399 | -10.17 | C_15_H_10_O_6_ | 175.0392, 151.0018, 133.0279 | kaempferol | A |
| 50 | 13.80 | 431.0983 | 431.1009 | -6.03 | C_21_H_19_O_10_ | 269.0451 | emodin-6-o-β-D-glucoside | I |
| 51 | 14.00 | 343.0835 | 343.0818 | 4.96 | C_18_H_16_O_7_ | 313.0328, 285.0362, 270.0164 | obtusin | I |
| 52 | 14.55 | 357.1014 | 357.0975 | 10.92 | C_19_H_18_O_7_ | 313.0279, 269.0085, 241.0153 | methyl Obtusin | I |
| 53 | 15.25 | 329.0652 | 329.0661 | -2.74 | C_17_H_14_O_7_ | 298.0123, 270.0167 | aurantio-obtusin | I |
| 54 | 15.46 | 461.0711 | 461.0720 | -1.95 | C_21_H_18_O_12_ | 285.0410, 267.0309, 175.0237, | scutellarin | J |
| 55 | 15.59 | 519.1512 | 519.1503 | 1.73 | C_25_H_28_O_12_ | 227.1255, 209.0870 | 6'-O-cinnamoyl-8-epikingisidic acid | B |
| 56 | 15.83 | 149.0595 | 149.0597 | -1.34 | C_9_H_8_O_2_ | 149.0597, 131.0492 | cinnamic acid | F |
| 57 | 16.12 | 461.0741 | 461.0720 | 4.55 | C_21_H_18_O_12_ | 279.0408, 285.0025 | kaempferol-3-O-glucuronide | A |
| 58 | 16.85 | 431.0962 | 431.0978 | -3.71 | C_21_H_20_O_10_ | 325.1591, 285.0389 | kaempferol 7-O-α-L-rhamnopyranoside | A |
| 59 | 17.27 | 285.0382 | 285.0399 | -5.96 | C_15_H_10_O_6_ | 175.0391, 151.0022, 133.0279 | luteolin | A,C |
| 60 | 17.50 | 269.0459 | 269.0450 | 3.35 | C_15_H_10_O_5_ | 151.0020, 149.0228, 117.0321 | apigenin | A |
| 61 | 18.12 | 539.1752 | 539.1765 | -2.41 | C_25_H_32_O_13_ | 377.1230, 275.0855 | oleuropein | B |
| 62 | 19.18 | 287.0551 | 287.0556 | -1.74 | C_15_H_12_O_6_ | 151.0025, 135.0438 | eriodictyol | J |
| 63 | 19.25 | 255.2331 | 255.2324 | 2.74 | C_16_H_32_O_2_ | 255.2331 | hexadecanoic acid | H |
| 64 | 19.57 | 455.3552 | 455.3525 | 5.93 | C_30_H_48_O_3_ | 455.3551, 201.0361 | oleanolic acid or ursolic acid | B, D |
| 65 | 19.70 | 131.0492 | 131.0497 | -3.82 | C_9_H_8_O | 131.0492 | cinnamaldehyde | F |
| 66 | 19.82 | 161.0594 | 161.0603 | -5.59 | C_10_H_10_O_2_ | 131.0493 | 2­methoxycinnamaldehyde | F |
| 67 | 20.50 | 413.3765 | 413.3783 | -4.35 | C_29_H_50_O | 413.3768 | β-sitosterol | H |
| 68 | 20.91 | 471.3503 | 471.3474 | 6.15 | C_30_H_48_O_4_ | 453.3360, 425.3397 | echinocystic acid | C |
| 69 | 21.20 | 271.0620 | 271.0606 | 5.16 | C_15_H_12_O_5_ | 151.0021, 119.0489 | naringenin | E |
| 70 | 21.25 | 502.1558 | 502.1566 | -1.59 | C_20_H_27_NO_11_ | 295.1047, 133.0652 | amygdalin | H |
| 71 | 21.73 | 411.3650 | 411.3627 | 5.59 | C_29_H_48_O | 411.3651, 397.3452 | stigmasterol | E |
| 72 | 22.71 | 181.0495 | 181.0501 | -3.31 | C_9_H_9_O_4_ | 151.6605, 136.9081 | syringaldehyde | F |

Note: A table from our previous published study using the same batch of JMT drug is provided for reference (W. Song *et al*., Jinmaitong, a Traditional Chinese Compound Prescription, Ameliorates the Streptozocin-Induced Diabetic Peripheral Neuropathy Rats by Increasing Sciatic Nerve IGF-1 and IGF-1R Expression, Frontiers in Pharmacology 10 (2019) 255. doi: 10.3389/fphar.2019.00255). A, seeds of *Cuscuta chinensis* Lam.; B, seeds of *Ligustrum lucidum* Ait.; C, whole herb of *Eclipta prostrata* L.; D, whole herb of *Prunella vulgaris* L.; E, seeds of *Litchi chinensis* Sonn.; F, tender stem of *Cinnamomum cassia* Presl.; G, rhizoma of *Corydalis yanhusuo* W.; H, seeds of *Prunus persica* L; I, seeds of *Cassia obtusifolia* L. or *Cassia tora* L.; J, radix and rhizoma of *Asarum heterotropiodes* F.
