# Supplementary material for: Jinmaitong ameliorates diabetic peripheral neuropathy in streptozotocin-induced diabetic rats by modulating gut microbiota and neuregulin 1
Source: Aging (Albany NY). 2020 Sep 13;12(17):17436–58. doi: 10.18632/aging.103750 (PMC7521543; doi:10.18632/aging.103750)
Supplement: Supplementary Table 3 [file aging-12-103750-s005..pdf]

SUPPLEMENTARY TABLE

Supplementary Table 3. Detailed information of the antibodies used in this study.

Name of Antibody	Manufacture (Host)	Working Dilution	Applications
Contactin-associated protein 1 (Caspr)	Abcam (Rabbit)	1:500	IF
Amyloid precursor protein (App)	Genetex (Rabbit)	1:500	IF
beta Tubulin 3 (βIII-tubulin)	Genetex (Mouse)	1:500	IF
Protein gene product 9.5 (PGP 9.5)	Genetex (Rabbit)	1:200	IF
Alexa Fluor 488 goat anti-rabbit highly cross-adsorbed IgG (H+L)	Invitrogen (Goat)	1:200	IF
Alexa Fluor 594 goat anti-rabbit highly cross-adsorbed IgG (H+L)	Invitrogen (Goat)	1:200	IF
Alexa Fluor 594 goat anti-mouse highly cross-adsorbed IgG (H+L)	Invitrogen (Goat)	1:200	IF
